# Early Outcomes of a Curvature-Guided Strategy for Dual-Branch Revascularization in Zone 1 TEVAR

**DOI:** 10.3390/jcm15103961

**Published:** 2026-05-21

**Authors:** Lei Zhang, Chang Shu, Rui Li, Dexiang Xia, Xin Li

**Affiliations:** 1 Department of Vascular Surgery, The Second Xiangya Hospital, Central South University, Changsha 410011, China; zhanglei2022@csu.edu.cn (L.Z.); shuchang@csu.edu.cn (C.S.); 238211146@csu.edu.cn (R.L.); 2 The Institute of Vascular Diseases, Central South University, Changsha 410011, China; 3 State Key Laboratory of Cardiovascular Diseases, Center of Vascular Surgery, Fuwai Hospital, National Center for Cardiovascular Diseases, Chinese Academy of Medical Science and Peking Union Medical College, Beijing 100037, China

**Keywords:** thoracic endovascular aortic repair, dual-branch revascularization, unibody single-branched endograft, physician-modified endograft, anatomy-guided strategy

## Abstract

**Objective:** To evaluate the feasibility and early outcomes of a curvature-guided strategy that guides dual-branch revascularization during Zone 1 Thoracic Endovascular Aortic Repair (TEVAR) based on whether the aortic pathology is predominantly located on the greater or lesser curvature of the arch. **Methods:** In this retrospective, descriptive study (February 2023–June 2024), 43 consecutive patients were included under a predefined anatomical protocol. Of these, 3 patients (7.0%) were lost to follow-up and were included in the analysis of baseline characteristics and perioperative outcomes. The remaining 40 patients constituted the per-protocol follow-up cohort. Pathologies predominantly on the aortic arch’s greater curvature (*n* = 21) were managed with a Castor single-branched stent-graft for the left subclavian artery (LSA) and a left common carotid artery (LCCA) chimney stent. Those on the lesser curvature (*n* = 22) received a physician-modified endograft (PMEG). The primary outcome was technical success; secondary outcomes included safety, branch patency, and reintervention. **Results:** The overall technical success rate was 97.7% (100% in the Castor-chimney cohort [21/21] vs. 95.5% in the PMEG cohort [21/22]). No perioperative stroke, spinal cord ischemia, or retrograde type A dissection occurred in either cohort. Two type II endoleaks were observed: one intraoperative in the Castor-chimney cohort and one during follow-up in the PMEG cohort. Among the 40 patients (20 per cohort) who completed a median follow-up of 22.5 months, freedom from aortic-related reintervention was 95% (38/40), with one reintervention occurring in each cohort. Branch patency was 100% (20/20) in the PMEG cohort, whereas it was 95% (one asymptomatic LSA occlusion) in the Castor-chimney cohort. **Conclusions:** The implementation of a curvature-guided protocol, which rationally matches endograft techniques to arch anatomy, suggests acceptable early safety and efficacy for complex Zone 1 TEVAR. This anatomy-driven framework offers a potential personalized approach to dual-branch revascularization and warrants prospective validation.

## 1. Introduction

Thoracic endovascular aortic repair (TEVAR) is the standard treatment for descending aortic pathologies [1]. Technical advances have extended their application to more proximal landing zones. For pathologies involving the distal aortic arch, Zone 1 TEVAR is being performed with increasing frequency. This procedure requires coverage of the left common carotid artery (LCCA) origin, thus creating the challenge of achieving a durable proximal seal while simultaneously maintaining perfusion to both the LCCA and the left subclavian artery (LSA) [2]. Zone 1 TEVAR carries ischemic risks attributable to both covered supra-aortic branches: LCCA coverage is a major risk factor for anterior circulation stroke, whereas LSA coverage has been associated with posterior circulation stroke and a potentially higher rate of spinal cord ischemia. An important consideration in LSA management is that spinal cord ischemia remains a concern, with accumulating but still inconclusive evidence supporting this risk. Recent meta-analyses suggest that routine LSA revascularization reduces the risk of spinal cord ischemia and posterior circulation stroke compared with coverage alone [3,4].

Various revascularization techniques have been developed, including surgical debranching procedures (e.g., carotid-carotid-subclavian bypass) and endovascular options such as parallel stents (chimney/periscope), fenestrations (in situ or physician-modified), and custom-made branched devices. Each technique presents inherent trade-offs: debranching offers a durable solution but at the cost of significant surgical trauma [5]; parallel stents are more readily available but risk gutter endoleak [6]; fenestrations allow for anatomic reconstruction but are technically demanding [7]; custom devices are ideal yet constrained by cost and manufacturing delay [8]. Although hybrid TEVAR with supra-aortic debranching remains a widely accepted approach, it is associated with substantial surgical exposure and may carry higher perioperative morbidity in high-risk patients. In contrast, the rationale for selecting a less invasive, anatomically tailored endovascular or hybrid strategy is to reduce surgical trauma while preserving cerebral and upper extremity perfusion. However, this array of options creates a clinical dilemma, as no meta-analysis has directly compared hybrid surgical debranching with total endovascular techniques for dual-branch revascularization, and in the absence of robust comparative evidence, technique selection often depends on surgeon preference rather than a standardized, anatomy-driven algorithm [9].

It is hypothesized that a more rational approach would involve aligning the repair strategy’s mechanical properties with the specific challenges posed by aortic arch geometry. Specifically, pathology location (greater or lesser curvature) may be a key determinant, as each site dictates distinct hemodynamic and mechanical environments that favor different technical solutions. The greater curvature, subject to higher wall tension, might benefit from strategies emphasizing strong axial fixation and minimized parallel graft interfaces [10]. Conversely, the steeper angulation of the lesser curvature may require techniques offering superior conformability and precise fenestration [11].

Based on this rationale, our institution adopted a novel curvature-guided strategy. For greater-curvature pathologies, we employed a hybrid approach using a Castor single-branched stent-graft for the LSA combined with a chimney stent for the LCCA. For lesser curvature pathologies, we preferred a physician-modified endograft (PMEG) for simultaneous LCCA and LSA revascularization. This strategy was designed to address the limitations of more established approaches by tailoring the revascularization technique to the anatomical characteristics of the aortic arch, thereby potentially improving technical success while minimizing procedure-specific complications. Thus, this study aims to describe our initial experience with this anatomically guided strategy, reporting its perioperative and early outcomes, thereby evaluating the overall feasibility and safety of the decision-making framework in clinical practice and laying the groundwork for future prospective comparative studies.

## 2. Methods

### 2.1. Patient Selection and Study Design

This was a single-center, retrospective, descriptive cohort study reporting on the implementation and outcomes of a predefined curvature-guided management strategy for Zone 1 TEVAR requiring dual-branch revascularization. The study protocol was approved by the institutional Ethics Review Board.

The records of all consecutive patients who underwent Zone 1 TEVAR with dual-branch revascularization between February 2023 and June 2024 were reviewed. A total of 62 patients were identified. Patients were managed according to a standardized clinical pathway; the detailed patient flow and disposition are reported in the Results. The indication for surgery was an aortic pathology requiring Zone 1 proximal landing and revascularization of both the LCCA and LSA. Both elective and emergency cases were included. In emergency situations (e.g., complicated Type B aortic dissection with malperfusion or contained rupture), the same anatomical stratification and treatment assignment were applied, provided that diagnostic-quality preoperative CTA was available for planning. After applying the inclusion and exclusion criteria, 43 patients were included in the study; the reasons for exclusion of the 19 patients are detailed in the Results.

The cornerstone of this pathway was anatomical stratification based on the predominant location of the aortic pathology and the intended proximal seal zone relative to the aortic arch curvature. This stratification served as the sole determinant for assigning the technical approach, an application of the principle of matching device mechanics to anatomical challenge.

Accordingly, within this framework, patients were treated via one of two technical pathways: (1) For greater curvature pathologies, a Unibody single-branched stent-graft was used for LSA revascularization combined with a LCCA chimney stent. (2) For lesser curvature pathologies, a PMEG was used for simultaneous LCCA and LSA revascularization.

Inclusion criteria were: (1) CTA-confirmed aortic pathology requiring TEVAR with a proximal landing zone in Zone 1; (2) Necessity for revascularization of both the LCCA and LSA; (3) Complete preoperative, operative, and follow-up data. Exclusion criteria were: (1) Pathologies with a landing zone in Zone 0 or Zone 2; (2) Anatomy unsuitable for either planned strategy (e.g., LSA occlusion precluding Castor branch cannulation, or proximal landing zone diameter/angulation exceeding instructions for use of the selected endograft); (3) Use of alternative techniques (e.g., in situ fenestration, custom-made devices) as the primary repair.

Indication for Zone 1 Landing: Zone 1 proximal landing was indicated when (1) the aortic pathology involved the origin of the LCCA or extended to within 15 mm of it, precluding a Zone 2 landing with an adequate (>20 mm) proximal seal zone; (2) the pathology was located on the greater or lesser curvature requiring coverage of the LCCA origin to achieve a healthy sealing zone; or (3) the LCCA was compromised by the disease process itself. These indications formed the general indication for surgery within the protocol and were applied uniformly to both elective and emergency procedures.

Anatomical Stratification Criterion: Pathology location was classified via standardized preoperative computed tomography angiography (CTA) assessment using centerline reconstruction and multiplanar reformation. The greater curvature was defined as the segment from the anterior midline (12 o’clock) of the proximal ascending aorta, along the outer curve, to the posterior midline (6 o’clock) of the proximal descending aorta. The lesser curvature comprised the corresponding inner segment. Pathology was assigned to a cohort if its center (focal lesions) or >50% of the extent requiring coverage (extended lesions) fell within that segment. To ensure objectivity, all CTA assessments were performed independently by two vascular surgeons. Inter-observer agreement was measured with Cohen’s kappa, and disagreements were resolved by consensus with a third senior surgeon.

### 2.2. Baseline Data and Anatomical Measurements

Demographic, clinical, and detailed anatomical data were systematically collected. This encompassed standard demographic and clinical variables (age, sex, comorbidities, aortic pathology type) as well as comprehensive anatomical measurements derived from preoperative CT angiography. Key morphological parameters quantified to characterize the aortic arch and proximal seal zone included: the Myla arch type classification; dominance of the left vertebral artery; the aortic arch α-angle (between the ascending aorta and the arch) and β-angle (between the arch and the descending aorta), measured via centerline analysis; the diameters of the LCCA and LSA origins; the distance from the anterior wall of the LCCA to the anterior wall of the aorta (anterior margin diameter) and from its posterior wall to the aortic wall (posterior margin diameter); and the distance from the posterior margin of the LCCA to the anterior margin of the LSA. Additional anatomical variables recorded to characterize the proximal seal zone included: proximal landing zone length (measured from the LCCA ostium to the intended proximal seal point along the greater curvature), aortic diameter at the proximal landing zone, presence of mural thrombus or circumferential calcification at the landing zone, and the angulation of the LCCA and LSA relative to the aortic arch (defined as the angle between the vessel centerline and the aortic centerline at the ostium).

### 2.3. Devices

#### 2.3.1. Devices for the Hybrid Strategy (Unibody Single-Branched Stent-Graft with Chimney)

The primary endograft was the Castor unibody single-branched stent-graft system (MicroPort Medical, Shanghai, China), selected for arch repair due to its fully covered design without proximal or distal bare springs. Its integrated, pre-cannulated side branch facilitates LSA revascularization.

For the parallel chimney component to revascularize the LCCA, the Fluency Plus covered stent (Bard, Murray Hill, NJ, USA) was preferred to minimize gutter endoleak risk. In cases of a severely calcified LCCA ostium, the Omnilink Elite bare-metal stent (Abbott Vascular, Abbott Park, IL, USA) was used selectively for its superior radial force. All chimney stents were oversized by 5–10% relative to the target vessel.

#### 2.3.2. Devices for the PMEG Strategy

The primary endograft was the Ankura™ thoracic stent-graft system (Lifetech Scientific, Shenzhen, China), selected for its stable deployment mechanism and distinct “∞”-shaped radiopaque marker and longitudinal “strengthen strut” which aided rotational orientation during fenestration.

Bridging stent selection was standardized: For the LCCA, a balloon-expandable bare-metal stent (Omnilink Elite, Abbott Vascular, Abbott Park, IL, USA) was used exclusively for its radial force. For the LSA, management was individualized: a balloon-expandable covered stent (Viabahn, Gore, Flagstaff, AZ, USA) was used when secure seal was required at the fenestration site due to incomplete fabric apposition or to seal a proximal LSA tear; in select cases with favorable anatomy defined by a ≥5 mm seal zone within the LSA, confirmed intraoperative patency without endoleak, and absence of a proximal LSA entry tear, a bridging stent was omitted to minimize implant material.

#### 2.3.3. Rationale for Device Selection

The selection of these specific device systems was guided by their mechanical and procedural characteristics relative to the anatomical requirements of each pathway, rather than by commercial availability alone. For the greater curvature pathway, the Castor unibody design with an integrated pre-cannulated side branch was chosen because it eliminates one parallel graft interface at the LSA origin, the site where conventional double-chimney configurations have been associated with gutter-related endoleak. The Fluency Plus covered stent was preferred for the LCCA chimney to minimize gutter endoleak risk, while the Omnilink Elite bare-metal stent was reserved for cases with severely calcified LCCA ostia where its superior radial force is advantageous. For the lesser curvature pathway, the Ankura thoracic stent-graft was selected as the base platform for physician modification owing to its distinctive “∞”-shaped radiopaque marker and longitudinal strengthened strut, which serve as reliable fluoroscopic landmarks for rotational orientation, an essential requirement for precise fenestration alignment in the angulated inner curvature. The Omnilink Elite balloon-expandable bare-metal stent was used exclusively for the LCCA fenestration because its high radial force maintains fenestration patency against the aortic wall.

It should be noted that dedicated commercial branched aortic arch stent-grafts (e.g., Terumo Relay branched platform, Artivion Nexus) were not yet approved or commercially available at our institution during the study period (February 2023–June 2024). The devices used in this study represent the locally available armamentarium for total-endovascular dual-branch revascularization. The objective of this study was not to compare specific commercial devices but to evaluate a curvature-guided strategy that systematically matches technique to anatomy using the tools at hand.

### 2.4. Endovascular Procedure

All interventions were performed in a hybrid operating room under general anesthesia.

**Figure 1:**
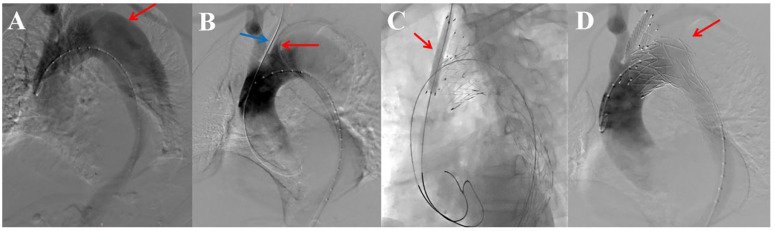
Key steps of the Castor single-branched stent-graft with LCCA chimney technique. (**A**) Preoperative angiography showing an arch aortic dissection (arrow) located on the greater curvature, requiring Zone 1 repair. (**B**) Schematic of the dual-wire setup: a brachio-femoral rail for the Castor graft (red arrow) and a separate wire from the surgical cutdown was advanced into the LCCA (blue arrow) for the chimney stent, positioned posteriorly to the main rail. (**C**) Fluoroscopic image illustrating post-deployment low-pressure balloon molding (arrow) within the chimney stent to optimize fabric apposition. (**D**) Completion angiography confirming successful exclusion of the dissection (arrow) and patency of both supra-aortic branches.

Castor branch in LSA + LCCA Chimney (Figure 1): Figure 1A shows the diagnostic angiography of a representative case. Vascular access was established via percutaneous puncture of the femoral artery and surgical cutdown of the left brachial artery and the LCCA. The core procedure involved establishing two parallel working channels. First, a brachio-femoral through-wire rail (Terumo Medical Corporation, Tokyo, Japan) was created for the delivery of the Castor stent-graft. Simultaneously, a separate Amplatz guidewire was advanced through the surgical cutdown of the LCCA to establish a dedicated track for the subsequent chimney stent deployment (Figure 1B). A critical preparatory step was to obtain an optimal fluoroscopic view confirming that the chimney guidewire coursed posterior to the primary delivery rail to minimize the risk of entanglement. The Castor stent-graft was deployed first, with its branch aligned at the origin of the LSA. This was followed by the deployment of the LCCA chimney stent. Immediate post-deployment balloon molding was performed exclusively within the chimney stent using a low-pressure balloon to optimize fabric apposition at the overlap zone, while care was taken to avoid compression of the adjacent Castor branch (Figure 1C). Completion angiography confirmed successful exclusion of the aortic pathology with preserved patency of both revascularized branches (Figure 1D).

**Figure 2:**
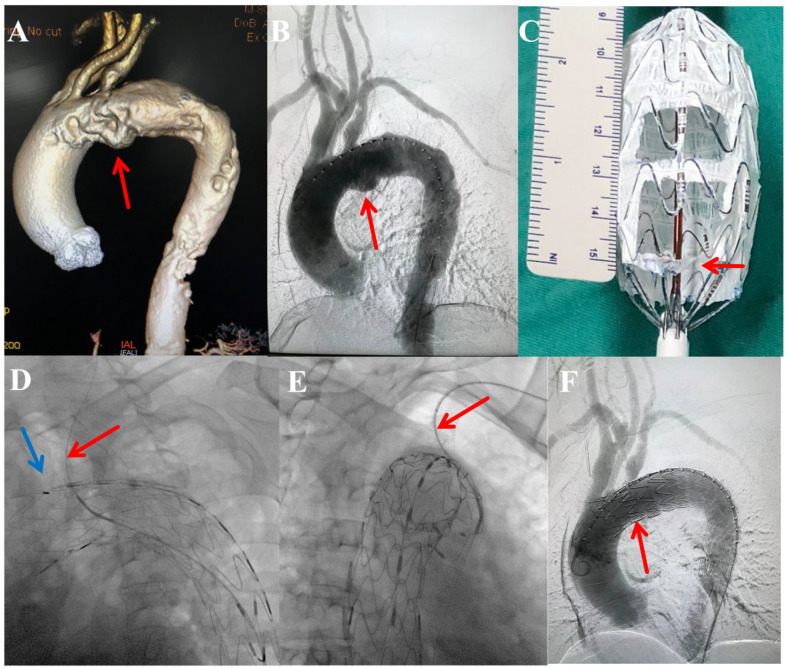
Key procedural steps of the physician-modified endograft (PMEG) technique. (**A**) Preoperative three-dimensional CTA reconstruction showing the aortic pathology and its relation to the supra-aortic branches (arrow). (**B**) Initial calibrated left anterior oblique angiography for pathology confirmation (arrow) and device sizing. (**C**) Illustration of back-table stent-graft modification: fenestrations for the LCCA and LSA (circles) are created distal and proximal to the “∞”-shaped marker (red arrow), respectively, and reinforced with radiopaque sutures. (**D**) During deployment under fluoroscopic guidance, the modified stent-graft is aligned as the “∞” marker appears straight (blue arrow); subsequently, a balloon-expandable bare-metal stent (red arrow) is deployed into the LCCA through the corresponding fenestration. (**E**) Fluoroscopic image showing subsequent deployment of a balloon-expandable covered stent (arrow) into the LSA when required for sealing. (**F**) Completion angiography confirming correct device position, exclusion of the aortic pathology, and patency of all supra-aortic branches (arrow).

PMEG for LCCA and LSA (Figure 2): Primary access was obtained via percutaneous femoral puncture. Additional access from the brachial or carotid artery was secured as required for target vessel cannulation. The procedure comprised the following steps: (1) Preoperative Anatomical Assessment: A dedicated three-dimensional volume-rendered reconstruction of the preoperative computed tomography angiography was systematically reviewed to characterize the aortic pathology, delineate its relationship to the supra-aortic branches, and confirm the anatomical indication for Zone 1 TEVAR with dual-branch revascularization (Figure 2A). (2) Initial Angiography: A calibrated left anterior oblique angiogram was performed to verify the preoperative CTA findings, confirm the absence of interval pathological changes, and establish the optimal working projection for device deployment (Figure 2B). (3) Stent-Graft Modification: An Ankura™ stent-graft was partially deployed on a sterile back table. Using electrocautery, two fenestrations were created. Their positions were individualized based on preoperative CTA measurements of the distances from the LCCA and LSA ostia to a fixed landmark (e.g., the distal end of the graft or the left subclavian artery). The LCCA fenestration was created distal to the “∞” shaped marker, and the LSA fenestration proximal to it. Both fenestrations were subsequently reinforced with a radiopaque suture before the device was re-sheathed (Figure 2C). (4) Device Alignment, Deployment, and Branch Revascularization: Under fluoroscopic guidance, the modified graft was introduced, rotationally aligned until the “∞” marker appeared straight and the “strengthened strut” lay along the greater curvature of the aortic arch, and then fully deployed. Subsequently, through sequential target vessel cannulation, a balloon-expandable bare-metal stent was deployed into the LCCA (Figure 2D). The LSA was managed in an individualized manner: a covered stent was placed when required to ensure a seal at the fenestration site or to exclude a primary entry tear in the proximal descending aorta that was not adequately covered by the main graft’s distal component (Figure 2E); otherwise, a fenestration-only strategy was employed in select cases with favorable anatomy (≥5 mm seal zone, no endoleak, no proximal tear). (5) Completion: Final angiography was performed to confirm correct device position, patency of the revascularized branches, and the absence of Type I or III endoleak (Figure 2F). In case of a minor (slow-filling) Type II endoleak, no immediate intervention was performed. All access sites were percutaneously closed with vessel closure devices.

### 2.5. Outcome Measures and Follow-Up Protocol

Outcome Measures: Perioperative and follow-up outcomes were assessed using standardized definitions. The primary outcome was technical success, defined as the simultaneous achievement of all four following criteria: (1) successful delivery and precise deployment of all intended stent-grafts; (2) absence of Type I or III endoleak on completion angiography; (3) maintained patency of all targeted supra-aortic branches (LCCA and LSA) at procedure end; and (4) successful primary exclusion of the aortic pathology without unplanned conversion to open surgery.

Secondary outcomes encompassed the following key domains: (1) Safety Outcomes: including 30-day and overall all-cause mortality, and procedure-related morbidity (e.g., stroke, spinal cord ischemia, myocardial infarction, access site complications); (2) Efficacy and Device-Related Outcomes: including the occurrence and type of any endoleak; stent migration (>10 mm); stenosis (>50%) or occlusion of revascularized branches; retrograde type A dissection; and distal stent-graft-induced new entry (dSINE); (3) Intervention Outcomes: including any aortic- or branch-related reintervention during follow-up.

Follow-up Protocol: A standardized protocol was required. Baseline whole-aorta CTA was obtained between postoperative days 5–7 for anatomic reference. Follow-up was scheduled at 1, 6, 12 and months and annually thereafter, each including clinical evaluation and whole-aortic CTA to assess stent-graft integrity, pathology evolution, branch patency, and endoleaks. Additional imaging could be performed based on clinical and imaging findings.

Standardized CTA Acquisition Protocol: All follow-up CTA examinations were performed using a third-generation dual-source CT scanner (SOMATOM Force, Siemens Healthineers, Forchheim, Germany). The standardized acquisition parameters were as follows: slice thickness, 0.75 mm with a 0.5 mm reconstruction interval; tube voltage, 100–120 kVp with automated tube current modulation; pitch, 0.6–0.8; gantry rotation time, 0.28 s. ECG-gated acquisition was not routinely employed for follow-up CTA unless specifically indicated for the evaluation of suspected Type A aortic pathology or coronary involvement; non-ECG-gated helical acquisition was used as the standard protocol. A bolus of 60–80 mL of non-ionic iodinated contrast medium (Iopromide, 370 mg iodine/mL; Ultravist, Bayer, Berlin, Germany) was injected at a rate of 4–5 mL/s into an antecubital vein, followed by a 40 mL saline flush. Image acquisition was triggered in the arterial phase using a bolus-tracking technique with the region of interest placed in the descending thoracic aorta at the level of the carina (threshold, 100 HU; delay, 6 s). The scan range extended from the thoracic inlet to the symphysis pubis. All CTA data were transferred to a dedicated post-processing workstation (Syngo.via, Siemens Healthineers) for multiplanar reconstruction and centerline analysis.

Antiplatelet Regimen: In the absence of dedicated guidelines for these specific hybrid and fenestrated arch repair techniques, a uniform regimen was adopted based on institutional practice: dual antiplatelet therapy (aspirin 100 mg + clopidogrel 75 mg daily) for 6 months, followed by lifelong aspirin (100 mg daily).

### 2.6. Data Analysis

All statistical analyses were performed using IBM SPSS Statistics (Version 27.0). Consistent with the descriptive and exploratory nature of this study, analyses focused on summarizing data rather than testing formal hypotheses between the two strategic pathways. Continuous data are presented as mean ± standard deviation or as median with interquartile range (IQR), based on distribution. Categorical data are reported as counts and percentages. To characterize the outcomes associated with each anatomical-technical pathway, key results are presented separately by group. No statistical comparisons (e.g., *p*-values) were performed between the two groups, as the study was designed to describe the outcomes of an anatomy-driven strategy, not to directly compare the techniques. Perioperative outcomes were analyzed for all 43 patients with complete in-hospital data. For the 3 patients lost to follow-up, in-hospital medical records (including operative notes, daily progress notes, discharge summaries, and the protocol-specified baseline postoperative CTA obtained on days 5–7) were fully available and reviewed. This baseline CTA confirmed technical success and the absence of immediate complications in all three patients. These records confirmed that all three patients were discharged alive with an uneventful postoperative course. Time-to-event outcomes (e.g., freedom from reintervention, branch patency) were analyzed for the 40 patients who completed at least one follow-up visit. The 3 patients lost to follow-up were censored at the date of discharge. Their vital status after discharge could not be ascertained. Time-to-event outcomes are illustrated with Kaplan–Meier curves to descriptively summarize the early experience for each pathway. This study was neither designed nor powered to detect differences in clinical outcomes between pathways; therefore, no comparative statistical tests (e.g., log-rank) were performed, and the curves are presented for descriptive purposes only.

## 3. Results

### 3.1. Patient Flow and Cohort Derivation

**Figure 3:**
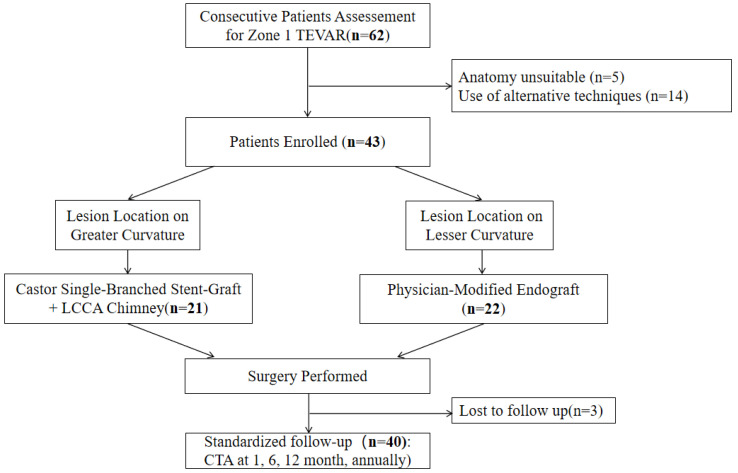
Clinical workflow of the curvature-based strategy for Zone 1 TEVAR with dual-branch revascularization.

The clinical workflow and patient disposition are illustrated in Figure 3. During the study period, 62 consecutive patients who underwent Zone 1 TEVAR with dual-branch revascularization were identified. After applying the inclusion and exclusion criteria, 43 patients were included. The reasons for excluding the remaining 19 patients were as follows: 5 patients (8.1% of the screened cohort) had aortic arch anatomy deemed unsuitable for either of the two protocol-defined techniques (e.g., extreme proximal landing zone angulation or diameter exceeding device instructions for use). Fourteen patients (22.6% of the screened cohort) were managed with alternative revascularization techniques not specified in the protocol, including in situ fenestration (*n* = 9) and custom-made branched endografts (*n* = 5). This choice was driven either by surgeon preference or by specific anatomical considerations outside the scope of the two predefined pathways. Consequently, 43 patients formed the study cohort and were stratified according to the curvature-guided strategy.

Of the 43 included patients, 3 (7.0%) were lost to follow-up (no scheduled visits or postoperative CTA after discharge). For these 3 patients, complete in-hospital medical records (including operative notes, daily progress notes, discharge summaries, and the protocol-specified baseline CTA on days 5–7) were reviewed. This baseline CTA confirmed technical success and the absence of immediate complications. All three underwent a technically successful procedure, had an uneventful perioperative course with no documented major adverse events, and were discharged alive in stable condition. Their available data up to the date of discharge were included in the analysis of baseline characteristics and perioperative outcomes. However, because no outpatient follow-up data were available, their long-term survival and graft-related outcomes could not be determined. The remaining 40 patients completed the scheduled follow-up protocol and formed the per-protocol cohort for time-to-event outcome analysis (e.g., branch patency and freedom from reintervention).

### 3.2. Patient Demographics and Baseline Anatomical Characteristics

Baseline characteristics for the overall cohort and each pathway are detailed in Table 1. The cohorts resulting from this anatomy-driven selection exhibited differences in the distribution of aortic pathology type and aortic arch type (Myla classification), reflecting inherent associations between disease entity and arch geometry rather than selection bias. As per the strategy’s design, all patients in the Castor-Chimney pathway had greater curvature involvement, and all in the PMEG pathway had lesser curvature involvement. Quantitative aortic arch angles (α and β) were comparable between the two pathways.

Additional anatomical characteristics relevant to proximal seal zone assessment are summarized in Table 1. The proximal landing zone length (measured from the LCCA ostium to the intended proximal seal point) was comparable between pathways, with a mean of 24.3 ± 3.8 mm in the Castor-chimney cohort and 23.9 ± 4.3 mm in the PMEG cohort. Aortic diameter at the landing zone was 32.5 ± 3.1 mm and 33.3 ± 3.5 mm, respectively. Mural thrombus at the landing zone was present in 4 patients (19.0%) in the Castor-chimney cohort and 7 patients (31.8%) in the PMEG cohort. LCCA takeoff angle relative to the aortic arch was 78.5° ± 12.2° and 82.0° ± 14.5°, respectively, while LSA takeoff angle was 85.6° ± 11.7° and 88.3° ± 13.2°.

### 3.3. Procedural and Perioperative Outcomes

Procedural outcomes are summarized in Table 2. Of the 43 patients enrolled, all had complete perioperative data and were included in the analysis. The overall technical success rate for the implemented strategy was 97.7% (42/43) (100% in the Castor-chimney cohort [21/21] vs. 95.5% in the PMEG cohort [21/22]). A single technical failure (intraoperative Type Ia endoleak) occurred in the PMEG pathway, which was successfully managed with a proximal aortic cuff. Procedural parameters differed between pathways as expected, given their distinct technical workflows: mean operative time was 99.8 ± 18.9 min for the Castor-Chimney pathway and 120.2 ± 35.5 min for the PMEG pathway. The mean length of descending aortic coverage, measured from the distal edge of the LSA ostium to the distal end of the stent-graft along the aortic centerline on the first postoperative CTA, was 158.4 ± 32.6 mm for the overall cohort (Castor-chimney: 155.6 ± 33.1 mm; PMEG: 161.0 ± 32.3 mm). No perioperative stroke, spinal cord ischemia, myocardial infarction, or access site complications requiring intervention occurred in either pathway. Access strategies differed as necessitated by the technical specifications of each approach. Within the PMEG pathway, LSA management was individualized: a bridging stent was placed in 8 cases (36.4%), while a “fenestration-only” approach was employed in 14 cases (63.6%) with favorable anatomy meeting the predefined criteria (≥5 mm seal zone within the LSA, confirmed intraoperative patency without endoleak, and absence of a proximal LSA entry tear). One intraoperative Type II endoleak was noted in the Castor-Chimney pathway.

### 3.4. Follow-Up and Early Term Outcomes

Of the 43 patients, 40 (93.0%) completed at least one follow-up visit with CTA and constituted the per-protocol follow-up cohort for time-to-event analyses. The 3 patients lost to follow-up (1 in the Castor-chimney pathway, 2 in the PMEG pathway) were censored at the date of hospital discharge. Their available in-hospital data confirmed uneventful perioperative courses and technically successful procedures.

The median follow-up for the entire cohort was 22.5 months (IQR: 16.3–26.8 months), with similar follow-up durations between pathways (Table 3). Given that the present study was designed to report early outcomes, the number of patients remaining under observation beyond 24 months was naturally limited. The actual loss-to-follow-up rate was low (4.8% in the Castor-chimney group and 9.1% in the PMEG group), and no definitive comparative conclusions should be drawn from these descriptive curves. No retrograde type A dissection or distal stent-graft-induced new entry (dSINE) occurred during follow-up. One new Type II endoleak was detected in the PMEG pathway. No Type I or III endoleaks were observed. One death from non-aortic causes (malignancy) occurred in the Castor-Chimney pathway at 11 months post-operation. Notably, in the PMEG subgroup managed with a “fenestration-only” approach for the LSA (*n* = 12 in the per-protocol cohort), there were no instances of branch compromise or fenestration-related endoleak. A summary of complications and events is provided in Table 3; data are presented to describe the outcome profile for each pathway separately, without inferential comparison.

Aortic remodeling was systematically assessed in all 40 patients who completed follow-up by comparing the first postoperative CTA (days 5–7) with the most recent follow-up CTA. Among the 20 TBAD patients, the mean true lumen diameter at the mid-descending thoracic level increased from 14.2 mm at baseline to 22.5 mm at the latest follow-up, indicating positive true lumen expansion. Complete false-lumen thrombosis in the thoracic segment was achieved in 14 patients (70.0%), partial thrombosis in 5 (25.0%), and a patent false lumen persisted in 1 (5.0%). No patient showed ≥5 mm expansion of the total aortic diameter. Among the 15 TAA patients with complete follow-up, the maximum aneurysm sac diameter remained stable (mean change −1.2 ± 3.4 mm), with sac regression ≥ 5 mm observed in 4 patients (26.7%) and no sac expansion ≥ 5 mm. All 5 TAU patients demonstrated complete resolution of the ulcer crater and associated intramural hematoma. These findings are summarized in Table 3.

**Figure 4:**
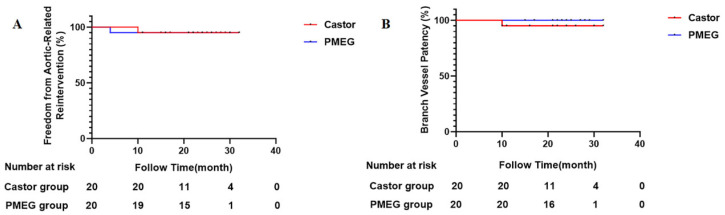
Kaplan–Meier analysis summarizing early outcomes. (**A**), Freedom from aortic-related reintervention by pathway (greater curvature: Castor-chimney, red; lesser curvature: PMEG, blue). (**B**), Branch vessel patency by pathway. A single LSA occlusion occurred in the greater curvature pathway at 10 months, while the lesser curvature pathway maintained 100% patency. No comparative statistical tests were performed, and the curves are presented for descriptive purposes only. The number of patients remaining at risk at 0, 10, 20,30 and 40 months for each pathway is shown in the table below.

Kaplan–Meier curves illustrating the early experience for freedom from aortic-related reintervention and branch vessel patency are presented in Figure 4A,B, respectively. Over the follow-up period, one aortic-related reintervention was recorded in each pathway (detailed in Table 3). Regarding branch patency, a single, asymptomatic LSA occlusion occurred in the Castor-Chimney pathway at 10 months, while branch patency remained 100% in the PMEG pathway throughout the observation period. In the 3 patients lost to follow-up, no adverse events were documented during their hospitalization; their vital status at the time of analysis could not be ascertained.

## 4. Discussion

This report describes the initial experience and outcomes of a curvature-based strategy for managing Zone 1 TEVAR with dual-branch revascularization. The strategy used the predominant location of the aortic pathology (greater vs. lesser arch curvature) as the sole criterion for selecting between two technical pathways: the Castor single-branched stent-graft with chimney for greater-curvature lesions, and the PMEG for lesser-curvature lesions. Within this framework, both approaches were associated with high technical success and favorable early outcomes. Notably, over a median follow-up of 22.5 months in the 40 patients who completed follow-up, no delayed Type I/III endoleaks, retrograde type A dissections, or dSINE events occurred, aligning with the goals of this anatomy-tailored approach.

Given the retrospective, non-randomized design of this study, it is crucial to underscore that treatment assignment was determined by the pre-existing anatomical feature of pathology location. Thus, the two cohorts exemplify the application of the strategy’s two distinct technical pathways to two distinct anatomical challenges. Consequently, data presentation is descriptive in nature, illustrating outcomes within each anatomical-technical context. Of the 43 patients included in the study, 3 (7.0%) were lost to follow-up and were excluded from time-to-event analyses; their available in-hospital data confirmed uneventful perioperative courses. The primary finding is that within this anatomy-guided strategy, both technical pathways yielded favorable early outcomes, supporting the feasibility of this decision-making framework.

The absence of Type Ic endoleak in the greater curvature pathway (Castor-chimney) was observed in this series, a finding that contrasts with reported rates for conventional double-chimney techniques [12,13]. One possible explanation is the ‘partial-eclipse’ configuration, where the chimney stent nests within the convex contour of the integrated Castor branch, potentially reducing gutter space, and the use of an integrated branch which eliminates one parallel graft interface [14,15]. This proposed biomechanical hypothesis, while consistent with our observations, remains speculative and warrants validation through computational modeling.

Regarding branch patency, the 100% patency rate at follow-up in the lesser curvature pathway (PMEG) is consistent with outcomes reported for physician-modified grafts from other centers, reinforcing the suitability of this technique within the strategy for precise anatomical reconstruction of lesser curvature pathologies [16,17]. Importantly, this includes the 12 patients (60% of the 20 PMEG patients who completed follow-up) in whom an unstented fenestration was utilized for the LSA. In the entire PMEG cohort of 22 patients, 14 (63.6%) were managed with the fenestration-only approach. This finding supports the concept that a precisely crafted fenestration, in highly selected anatomy (≥5 mm seal zone within the LSA, confirmed intraoperative patency without endoleak, and absence of a proximal LSA entry tear), can serve as an effective and durable means of branch preservation without mandatory stent placement, thereby reducing implant burden and potential long-term stent-related complications. However, this “fenestration-only” strategy remains exploratory and should be applied with strict adherence to the defined anatomical criteria until further validation is available. The delayed LSA occlusion in the Castor cohort may be attributed to a late healing response initiated by intraoperative injury. Microtrauma at the ostium caused by balloon dilation likely triggered a chronic pathological remodeling process dominated by neointimal hyperplasia, ultimately resulting in occlusion at 10 months. This sequence underscores that iatrogenic manipulative injury remains a potential threat to long-term patency even with branched stent-graft designs, warranting particular attention in the complex hemodynamic environment of the aortic arch. The absence of events for major safety endpoints (e.g., stroke, spinal cord ischemia) in both pathways is reassuring and suggests that this stratified strategy can be executed safely in experienced, high-volume centers [18,19].

Several dedicated branched aortic arch stent-grafts have received regulatory approval in other regions (e.g., the Terumo Relay branched platform and the Artivion Nexus off-the-shelf single-branched device). These commercial systems offer the advantage of standardized manufacturing and potentially simplified implantation workflows, and future comparisons between our curvature-guided strategy and such dedicated devices, once they become broadly available, would be valuable. However, it must be emphasized that the present study addresses a practical clinical question in a setting where these commercial arch-branched devices were not accessible at the time: given the available endovascular tools, can an anatomy-driven choice between two distinct technical pathways improve early outcomes? Our findings suggest that a rational matching of device mechanics to the distinct anatomical challenges posed by greater versus lesser curvature pathology can yield acceptable early results. Whether a dedicated commercial single-branched device would perform equivalently or superiorly to either pathway in specific anatomical scenarios remains an important question for future prospective investigation.

An important anatomical insight from this study is that the key quantitative aortic arch parameters (α-angle, β-angle) were similar between the two pathways. This indicates that “lesion location on the greater or lesser curvature” and “overall aortic arch tortuosity” are separable anatomical variables, supporting the premise of using curvature as an independent selection criterion. Furthermore, the observed trends in pathology distribution (e.g., higher TBAD proportion in the lesser curvature/PMEG cohort) suggest that curvature location may also correlate with specific disease entities. Collectively, this strengthens the internal rationale for the curvature-based stratification and reinforces that the subsequent outcome profiles are linked to distinct anatomy-pathology-technology complexes, which the strategy aims to optimally align.

Based on the institutional experience reflected in this study, the following clinical workflow, which operationalizes the curvature-based strategy, has been conceptualized for aortic pathologies involving Zone 1 and requiring revascularization of both the LCCA and LSA. First, a determination is made whether the lesion predominantly involves the greater or lesser curvature of the aortic arch through meticulous CTA analysis. For pathologies primarily located on the greater curvature, the Castor single-branched stent-graft with LCCA chimney technique is the preferred approach within this strategy, based on its procedural characteristics and the observed immediate and early seal integrity in the present series, which may be aided by the “partial-eclipse” configuration. For pathologies primarily located on the lesser curvature, the physician-modified fenestrated stent-graft technique is preferred within this strategy for its superior anatomical conformability and branch alignment, which better accommodates the acute angulation, and has demonstrated acceptable branch patency in this and other reports [20]. It is important to emphasize that the implementation of this strategy and its associated pathways is inherently dependent on the operator’s proficiency in both techniques, and its general applicability requires further evaluation in prospective, multicenter settings.

This study has several inherent limitations. Its retrospective, single-center, non-randomized design, while reflective of real-world clinical decision-making, cannot eliminate selection bias or unmeasured confounding. As detailed above, we report the outcomes associated with two distinct anatomy-technology pairings as per the strategy rather than a direct, randomized comparison of the techniques. Furthermore, the two cohorts inherently differed in pathology type and arch morphology, reflecting the natural association between these factors and curvature location; these differences preclude any direct comparison of technique efficacy. Importantly, the current strategy and its associated technical pathways were applied in a high-volume center with expertise in both Castor-chimney and PMEG techniques; the generalizability of these findings to lower-volume centers or operators with limited experience in one of the two techniques remains to be established. Although the sample size of 43 patients (40 with complete follow-up) is considerable for a single-center study, it remains underpowered to detect differences in rare but significant events or to perform adequately adjusted analyses for potential confounders. Therefore, any observed numerical differences between pathways should be interpreted as descriptive findings within this strategic framework. A median follow-up of 22.5 months, while adequate for assessing early outcomes, remains insufficient to fully evaluate the long-term durability of these aortic repairs [21,22]. The heterogeneity in LSA management within the PMEG pathway may also introduce confounding. Furthermore, the 3 patients (7.0%) lost to follow-up represent a potential source of attrition bias, as their long-term outcomes could not be ascertained. Moreover, while we have added detailed anatomical variables (landing zone length, aortic diameter, mural thrombus, branch angulation) to better characterize the cohorts, the limited sample size precludes meaningful stratification or adjusted analyses to assess their independent impact on outcomes. Additionally, given the relatively young mean age of the cohort and the high proportion of TBAD patients, the presence of undiagnosed connective tissue disorders, a relative or absolute contraindication for elective endovascular repair, cannot be definitively excluded, as systematic genetic testing was not performed. In addition, the optimal antiplatelet regimen following these complex arch reconstructions remains undefined, and our institutional DAPT protocol, while uniformly applied, lacks procedure-specific evidence; dedicated studies are needed to determine the ideal agent(s), duration, and risk-benefit profile in this population. Finally, the curvature-based anatomical stratification carries inherent subjectivity in extended or circumferential pathologies; although high inter-observer agreement (κ = 0.87) supports its reproducibility, borderline cases remain a challenge that future refinements with semi-automated quantitative tools may help address. These limitations clearly indicate that the current observations and the proposed curvature-based strategy and clinical workflow require further validation in prospective, multicenter registries or randomized trials with extended follow-up.

In conclusion, this retrospective analysis of our initial experience with a curvature-based clinical strategy suggests acceptable early safety and efficacy profiles for Zone 1 TEVAR with dual-branch revascularization. By systematically matching specific techniques (Castor-chimney, PMEG) to specific anatomical challenges (greater and lesser curvatures), this approach provides a potential rational framework for personalizing therapy. The findings warrant further prospective investigation to validate and refine this anatomy-driven decision-making paradigm.

## Figures and Tables

**Table 1:** Epidemiological Characteristics and Comorbidities.

Variables	Total (*n* = 43)	Castor (*n* = 21)	PMEG (*n* = 22)
**Demographics**			
Age, y	62.5 ± 11.3	62.9 ± 10.6	62.1 ± 12.0
Male, *n* (%)	33 (76.7)	17 (81.0)	16 (72.7)
**Disease Type**			
TBAD, *n* (%)	22 (51.2)	8 (38.1)	14 (63.6)
TAA, *n* (%)	16 (37.2)	11 (52.4)	5 (22.7)
TAU, *n* (%)	5 (11.6)	2 (9.5)	3 (13.6)
**Personal history**			
Hypertension, *n* (%)	36 (83.7)	15 (71.4)	21 (95.5)
Diabetes mellitus, *n* (%)	3 (7.0)	2 (9.5)	1 (4.5)
CHD, *n* (%)	6 (14.0)	4 (19.0)	2 (9.1)
COPD, *n* (%)	2 (4.7)	0 (0.0)	2 (9.1)
Stroke, *n* (%)	5 (11.6)	1 (4.8)	4 (18.2)
Alcohol excess, *n* (%)	5 (11.6)	3 (14.3)	2 (9.1)
Smoking, *n* (%)	24 (55.8)	9 (42.9)	15 (68.2)
**Type of arch (Myla)**			
I, *n* (%)	27 (62.8)	11 (52.4)	16 (72.7)
II, *n* (%)	15 (34.9)	10 (47.6)	5 (22.7)
III, *n* (%)	1 (2.3)	0 (0.0)	1 (4.5)
**Left vertebral artery**			
Dominant	17 (39.5)	9 (42.9)	8 (36.4)
Isolated	0 (0.0)	0 (0.0)	0 (0.0)
**Lesion Location**			
Greater Curvature, *n* (%)	21 (48.8)	21 (100)	0 (0.0)
Lesser Curvature, *n* (%)	22 (51.2)	0 (0.0)	22 (100)
**Aortic Arch Geometry, degree**			
α-angle	98.6 ± 15.1	96.9 ± 14.8	100.2 ± 15.8
β-angle	132.5 ± 18.5	131.0 ± 17.2	133.9 ± 20.0
**Proximal Seal Zone Anatomy**			
* Proximal landing zone length, mm	24.1 ± 4.1	24.3 ± 3.8	23.9 ± 4.3
Aortic diameter at landing zone, mm	32.9 ± 3.3	32.5 ± 3.1	33.3 ± 3.5
Mural thrombus at landing zone, *n* (%)	11 (25.6)	4 (19.0)	7 (31.8)
Circumferential calcification at landing zone, *n* (%)	5 (11.6)	3 (14.3)	2 (9.1)
LCCA ostium involvement by pathology, *n* (%)	13 (30.2)	5 (23.8)	8 (36.4)
**Branch Takeoff Angle, degree**			
LCCA takeoff angle	80.3 ± 13.4	78.5 ± 12.2	82.0 ± 14.5
LSA takeoff angle	87.0 ± 12.4	85.6 ± 11.7	88.3 ± 13.2

Abbreviations: TBAD, type B aortic dissection; TAA, thoracic aortic aneurysm; TAU, thoracic aortic ulcer; CHD, coronary heart disease; COPD, chronic obstructive pulmonary disease * *Proximal landing zone length was defined as the distance from the proximal edge of the LCCA ostium to the proximal margin of the aortic pathology along the greater curvature on centerline reconstruction. This length quantifies the disease-free aortic wall available for proximal sealing within Zone 1 and is distinct from the short pathology-to-LCCA distance that necessitated a Zone 1 landing.*

**Table 2:** Procedural and perioperative outcomes by treatment pathway.

Variables	Total (*n* = 43)	Castor (*n* = 21)	PMEG (*n* = 22)
**Procedural Outcomes**			
Technical success, *n* (%)	42 (97.7)	21 (100.0)	21 (95.5)
Operation time, min	110.2 ± 30.2	99.8 ± 18.9	120.2 ± 35.5
Descending aortic coverage length, mm	158.4 ± 32.6	155.6 ± 33.1	161.0 ± 32.3
**Anatomical Parameters, mm**			
LCCA anterior margin diameter	33.0 ± 2.3	32.6 ± 2.1	33.3 ± 2.5
LCCA posterior margin diameter	31.8 (30.6, 34.2)	31.8 (30.5, 34.2)	31.7 (30.8, 35.5)
LCCA diameter	7.1 (6.9, 7.8)	6.9 (6.8, 7.2)	7.2 (6.9, 8.5)
LSA diameter	8.6 ± 0.9	8.7 ± 1.0	8.5 ± 0.9
LCCA posterior margin to LSA anterior margin	5.9 (4.4, 8.4)	5.7 (4.4, 8.3)	5.9 (4.2, 8.5)
**Access Route, *n* (%)**			
Femoral artery	43 (100.0)	21 (100.0)	22 (100.0)
Brachial artery	21 (48.8)	21 (100.0)	0 (0.0)
Carotid artery	24 (55.8)	21 (100.0)	3 (13.6)
**Surgical methods, *n* (%)**			
Castor + chimney stent	21 (48.8)	21 (100.0)	0 (0.0)
Fenestration + LCCA stent	22 (51.2)	0 (0.0)	22 (100.0)
Fenestration + LCCA + LSA stents	8 (18.6)	0 (0.0)	8 (36.4)
**Branch Stent Type, *n* (%)**			
LCCA bare stent	25 (58.1)	3 (14.3)	22 (100.0)
LCCA covered stent	18 (41.9)	18 (85.7)	0 (0.0)
LSA bare stent	0 (0.0)	0 (0.0)	0 (0.0)
LSA covered stent	29 (67.4)	21 (100.0)	8 (36.4)
**Perioperative Safety Outcomes, *n* (%)**			
Stroke	0 (0.0)	0 (0.0)	0 (0.0)
Spinal cord ischemia	0 (0.0)	0 (0.0)	0 (0.0)
Myocardial infarction	0 (0.0)	0 (0.0)	0 (0.0)
Access site complication	0 (0.0)	0 (0.0)	0 (0.0)
**Intraoperative complication, *n* (%)**			
Type I endoleak	1 (2.3)	0 (0.0)	1 (4.5)
Type II endoleak	1 (2.3)	1 (4.8)	0 (0.0)
Type III endoleak	0 (0.0)	0 (0.0)	0 (0.0)

**Table 3:** Complications and events during follow-up.

Variables	Total (*n* = 40)	Castor (*n* = 20)	PMEG (*n* = 20)
Median follow-up time, m	22.5 (16.3, 26.8)	21.5 (16.0, 25.8)	23.5 (21.3, 27.8)
**Aortic remodeling outcomes**			
TBAD patients, *n*	20	8	12
True lumen increase, mm	8.3 ± 3.1	7.9 ± 3.3	8.5 ± 3.0
Complete FL thrombosis, *n* (%)	14 (70.0)	5 (62.5)	9 (75.0)
TAA sac regression ≥5 mm, *n* (%)	4 (26.7)	2 (28.6)	2 (25.0)
TAU ulcer resolution, *n* (%)	5 (100)	2 (100)	3 (100)
Retrograde dissection, *n* (%)	0 (0.0)	0 (0.0)	0 (0.0)
Endoleak, *n* (%)	1 (2.5)	0 (0.0)	1 (5.0)
Branch stent stenosis or occlusion, *n* (%)	1 (2.5)	1 (5.0)	0 (0.0)
dSINE, *n* (%)	0 (0.0)	0 (0.0)	0 (0.0)
All Stent-related reintervention, *n* (%)	2 (5.0)	1 (5.0)	1(5.0)
All reintervention	2 (5.0)	1 (5.0)	1(5.0)
All-cause death, *n* (%)	1 (2.5)	1 (5.0)	0 (0.0)

Abbreviation: dSINE, distal stent graft-induced new entry.

## Data Availability

The data underlying this article can be shared upon reasonable request to the corresponding author.
